# Deploying deep Solanaceae domestication and virus biotechnology knowledge to enhance food system performance and diversity

**DOI:** 10.1093/hr/uhae205

**Published:** 2024-07-27

**Authors:** Fabio Pasin, Mireia Uranga, Raghavan Charudattan, Choon-Tak Kwon

**Affiliations:** Instituto de Biología Molecular y Celular de Plantas (IBMCP), Consejo Superior de Investigaciones Científicas – Universitat Politècnica de València (CSIC-UPV), 46011 Valencia, Spain; Laboratory for Plant Genetics and Crop Improvement, Division of Crop Biotechnics, Department of Biosystems, KU Leuven, 3001 Heverlee, Belgium; KU Leuven Plant Institute (LPI), KU Leuven, 3001 Heverlee, Belgium; Plant Pathology Department, University of Florida, 32609 Gainesville, FL, USA; BioProdex, Inc., 32609 Gainesville, FL, USA; Graduate School of Green-Bio Science, Kyung Hee University, 17104 Yongin, Republic of Korea; Department of Smart Farm Science, Kyung Hee University, 17104 Yongin, Republic of Korea

## Abstract

Our knowledge of crop domestication, genomics, and of the plant virosphere unevenly represents the taxonomic distribution of the global biodiversity, and, as we show here, is significantly enriched for the Solanaceae. Within the family, potato, tomato, eggplant, pepper, and over 100 lesser-known edible species play important nutrition and cultural roles in global and local food systems. Technologies using engineered viruses are transitioning from proof-of-concept applications in model plants to the precise trait breeding of Solanaceae crops. Leveraging this accumulated knowledge, we highlight the potential of virus-based biotechnologies for fast-track improvement of Solanaceae crop production systems, contributing to enhanced global and local human nutrition and food security.

## Introduction

Human food supply relies on a small fraction of global biodiversity. Among over 370 000 plant species, only ~1000 are cultivated for human nutrition or livestock feeding, and, at a global scale, ~30 crops directly or indirectly provide 95% of the calories we consume [1, 2]. Agricultural homogenization and narrow genetic diversity of modern crops weaken global food security and sustainability [3, 4], and are becoming a bottleneck for the release of improved varieties addressing the ever-growing demands for human nutrition. Diversity and resilience of our food systems may be enhanced by promoting underutilized crops with desirable features, such as stress resistance, and adaptation to local environments and low-input production settings [4–6].

The Solanaceae family comprises ~2600 species; these include potato (*Solanum tuberosum*), tomato (*Solanum lycopersicum*), eggplant (*Solanum melongena*), and sweet/chili pepper (*Capsicum annum*), which collectively contribute to global provisions. Over 100 lesser-known Solanaceae species [7] play important nutrition and cultural roles in food systems of local areas in Africa, Central and South America, and Asia, but the limited availability of resources and biotechnological tools has traditionally constrained their genetic improvement [8].

Plant viruses are a scientific treasure trove of valuable tools for improving crop systems [9]. Besides being a source of a variety of genetic elements and enzymes for biotechnology and synthetic biology, viruses can be repurposed *in toto* for delivering exogenous sequences into eukaryotic cells [10]. Recombinant virus technologies (RVTs) have enabled functional genomics and, more recently, the transient or heritable reprogramming of plant traits [11].

The Solanaceae has a prominent role in the study of viruses and the development of virus-based biotechnologies. Systematic disease characterization of solanaceous crops led to the discovery of viruses as infectious agents and the establishment of virology as a research discipline. RVTs are transitioning from proof-of-concept methodical studies in model species to the precise breeding of Solanaceae crop traits, as supported by recent application of virus systems for transient trait reprogramming [12], and heritable CRISPR–Cas-mediated editing of genomic loci of tomato, potato, pepper, and eggplant [13–15].

Our knowledge of crop domestication, genomics, and of the plant virosphere unevenly represents the taxonomic distribution of the global biodiversity [2, 16, 17], and, as we show here, is significantly enriched for the Solanaceae. By combining this cumulative stock of knowledge, we highlight the RVTs potential for fast-track improvement of Solanaceae crop production systems, leading to enhanced food security and diversity.

### Solanaceae crops for global and local food systems

**Figure 1:**
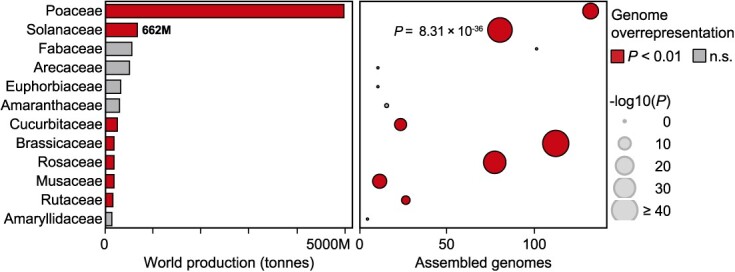
Overrepresentation of Solanaceae genomic resources. For each crop family, number of species with available genome assemblies as of December 2023 are shown (right), alongside global production (left; see Table S1 of Supplementary data); families whose genomic resources are significantly overrepresented based on species richness are indicated (Table S2).

The Solanaceae includes major crops, with potatoes, tomatoes, eggplants, and peppers, collectively contributing to 662 million tonnes in 2022 (Fig. 1). (Pan)genomic sequencing efforts aimed at assisting the breeding of these global food sources have recently acquired a more ample dimension. Among crop families and based on species richness, the Solanaceae is second only to the Brassicaceae in terms of being overrepresented in assembled genomes (*P* = 8.31 × 10^−36^; Fig. 1). These encompass cultivated varieties and landraces of global crops [18–21], as well as a number of lesser-known edible species [22, 23].

**Figure 2:**
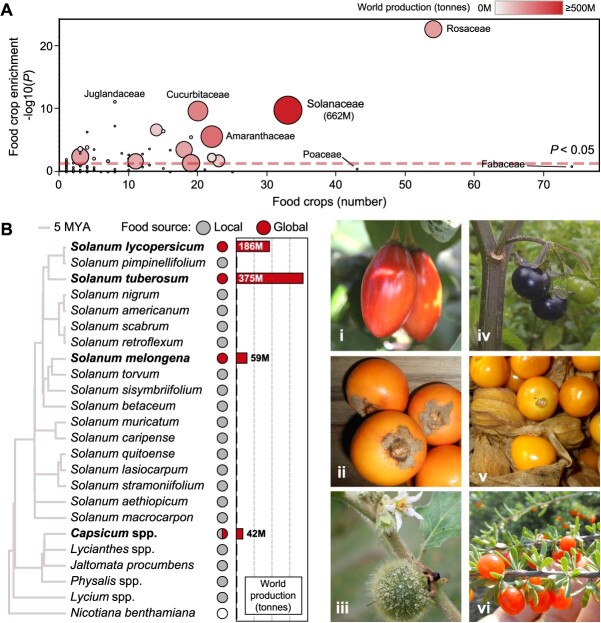
Overrepresentation of Solanaceae species used for food**.** (**A**) Non-random representation of plant families cultivated for human food. Food crop numbers and enrichment values based on species richness are shown for each family; global production is shown for families with a significant food crop enrichment (Table S3). (**B**) Solanaceae sources of global and local food. Left, phylogeny and world production of global Solanaceae crops is shown; key edible species with available genomic or transcriptomic resources (Table S4) are shown alongside *Nicotiana benthamiana*, a model plant used for RVTs development. Right, a range of local food sources: *Solanum betaceum* (i), *Solanum quitoense* (ii), *Solanum stramoniifolium* (iii), *Jaltomata procumbens* (iv), *Physalis peruviana* (v), *Lycium berlandieri* (iv); picture credits: rarepalmseeds, www.rarepalmseeds.com, reproduced with permission.

Underutilized solanaceous crops are an important resource for food system diversification. Among plant families with domesticated members and based on species richness, the Solanaceae is statistically overrepresented in species used for human food (*P* = 1.49 × 10^−10^; Fig. 2A), being the third most overrepresented group, below Rosaceae and Juglandaceae. By harnessing knowledge of the Solanaceae domestication genetics and available genomic resources, local human nutrition could be improved by accelerated breeding of underutilized crops to enhance the production of sweet fruits—such as tree tomato (*Solanum betaceum*), pepino (*Solanum muricatum*), lulo (*Solanum quitoense*)—vegetables—such as scarlet and gboma eggplants (*Solanum aethiopicum* and *Solanum macrocarpon*), terung asam (*Solanum lasiocarpum*)—or food with high levels of nutraceuticals—such as goji (*Lycium* spp.; Fig. 2B).

**Table 1:** Tomato knowledge for genetic improvement of underutilized food sources

**Trait**	**Tomato gene**		**Food source** ^**a**^
Plant architecture	Solyc06g074350	*SELF-PRUNING (SP)*	Currant tomato [26,27] Groundcherry [28,29]
	Solyc05g053850	*SELF-PRUNING 5G (SP5G)*	Currant tomato [27] Groundcherry [28]
	Solyc08g061560	*ERECTA (ER)*	Groundcherry [29]
Leaf/flower shape	Solyc01g010970	*ARGONUATE 7 (AGO7)*	Groundcherry [28]
Fruit number	Solyc02g077390	*MULTIFLORA (MULT)*	Currant tomato [26]
Fruit abscission	Solyc03g114840	*JOINTLESS 2 (J2)*	Groundcherry [28]
Fruit shape/size	Solyc02g085500	*OVATE (OV)*	Currant tomato [26] Groundcherry [28]
	Solyc10g076180	*SUPPRESSORS OF OVATE 1 (SOV)*	Groundcherry [28]
	Solyc04g081590	*CLAVATA 1 (CLV1)*	Groundcherry [28]
	Solyc04g056640	*CLAVATA 2 (CLV2)*	Groundcherry [28]
	Solyc11g071380	*CLAVATA 3 (CLV3)*	Currant tomato [27]
	Solyc02g083950	*WUSCHEL (WUS)*	Currant tomato [27]
Fruit quality/toxicity	Solyc10g079480	*LYCOPENE BETA CYCLASE (LCY-B)*	Groundcherry [28]
	Solyc04g040190	*LYCOPENE BETA CYCLASE 1 (LCY-B1)*	Currant tomato [26]
	Solyc02g091510	*GDP-L-GALACTOSE PHOSPHORYLASE 1 (GGP1)*	Currant tomato [27]
	Solyc02g069490	*STEROL SIDE CHAIN REDUCTASE 2 (SSR2)*	[30]
	Solyc01g090340	*GLYCOALKALOID METABOLISM 9 (GAME9)*	[31]
	Solyc06g074090	*7-DEHYDROCHOLESTEROL REDUCTASE 2* (7-DR2)	[32]
Mechanization	Solyc08g061910	GT-2-like transcription factor (*FS8.1*)	[33]

a Reported or potential use

Some of these exhibit a perennial habit that enhances the sustainability of agricultural systems by optimizing water and nutrient utilization [24], which is however linked to a variety of semi- or un-domesticated traits. *De novo* domestication strategies harnessing the genetic insights gained from model crops can enable the improvement of wild and underutilized relatives to breed varieties with compact plant architecture, shorter juvenile stages, increased fruit yield and size, synchronized ripening and fruits suitable for mechanical harvesting (Table 1), which would boost productivity per unit of land, preserve natural ecosystems, and reduce labor demands [5, 25].

For instance, currant tomato (*Solanum pimpinellifolium*) produces edible fruits and shows high resistance to salt and bacterial diseases; however, the small-sized fruits, suboptimal growth and flowering habits constrain its large-scale cultivation. CRISPR–Cas editing of *SELF-PRUNING (SP)*, *SELF-PRUNING 5G* (*SP5G*), *MULTIFLORA (MULT), OVATE (OV), LYCOPENE BETA CYCLASE 1 (LCY-B1),* and *GDP-L-GALACTOSE PHOSPHORYLASE 1 (GGP1)* orthologues allowed to obtain currant tomato lines with improved growth and flowering habits, and large fruits with high lycopene and vitamin C levels [26, 27].

Similarly*,* many *Physalis* species are locally consumed for their fruits and have high potential to aid global food supply, but there are barriers to their wider adoption (Fig. 2B). Breeding of loss-of-function alleles of *SP*, *SP5G* and *CLAVATA 1 (CLV1)* resulted in improved groundcherry (*Physalis grisea*) plants with compact plant architecture, and large flower number and fruit size [28]. *ERECTA* (*ER*) orthologues control internode length in tomato and groundcherry [29]; *ER* mutagenesis generated hypercompact groundcherry plants that are compatible with urban farming and show good productivity traits, including high fruit number [29].

### Virus discovery and biotechnology development in the Solanaceae

**Figure 3:**
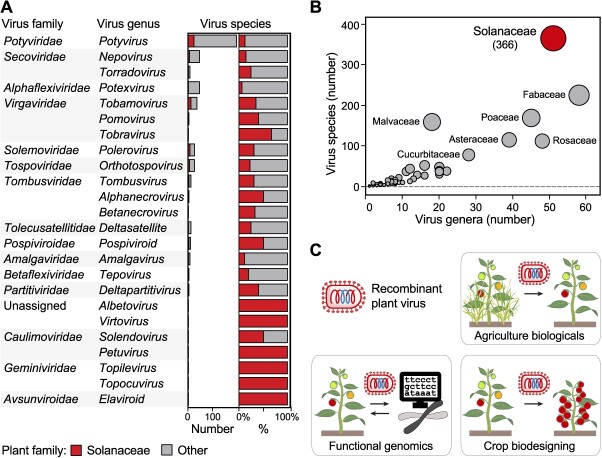
Representation of viral agents discovered in Solanaceae hosts, and applications of recombinant virus technologies (RVTs). (**A**) Virus discoveries in the Solanaceae contribute to understanding the plant virome diversity. Virus species recognized by the International Committee for the Taxonomy of Viruses (ICTV) were assigned to the host family of first discovery; virus genera whose reference members were discovered in Solanaceae hosts are shown alongside the total numbers of recognized virus species; for each genus, number and percentage of species first discovered in Solanaceae hosts are shown (Table S5). (**B**) Overrepresentation of the Solanaceae in current plant virosphere knowledge. Total numbers of ICTV plant virus species and genera per host family are plotted (Table S6). Solanaceae, a significant outlier based on virus species richness (*P* < 0.01), is highlighted; in parentheses, virus species number. (**C**) Agricultural applications of engineered plant viruses. RVTs allow accelerated functional genomics, implementation of biological agents (biologicals) for crop production and management, as well as crop biodesigning by transient or hereditable trait reprogramming.

Systematic disease characterization of Solanaceae crops has greatly contributed to our current understanding of viral diversity. Viral agents first discovered in Solanaceae hosts belong to divergent viral taxa, representing over 20 virus genera and catalyzing subsequent research (Fig. 3A). For example, potato virus Y (PVY) was the first discovered member of the *Potyvirus* genus, the largest group of plant RNA viruses [34], and was instrumental in the identification of viruses from other host families, which now account for 85% of the *Potyvirus* species richness (Fig. 3A). Similarly, viruses discovered in non-Solanaceae hosts now account for ~95% of the species richness of the *Potexvirus* genus, which was established upon discovery of potato virus X (PVX). As of December 2023, characterization of the Solanaceae virome lead to the first discovery of 366 virus species, assigned to one orphan and 51 recognized genera, which supports the significant contribution (*P* < 0.01) of the Solanaceae in the current plant virosphere knowledge (Fig. 3B).

This acquired knowledge spurred the development of multiple biotechnological applications (Fig. 3C). Since 2015, mild isolates of pepino mosaic virus (PepMV; genus *Potexvirus*) are commercialized as cross-protecting agents that safeguard greenhouse tomatoes from more severe infections [35]. In the same year, a natural isolate of tobacco mild green mosaic virus (TMGMV; genus *Tobamovirus*) was registered as a bioherbicide for the control of the invasive weed tropical soda apple (*Solanum viarum*) [36].

*Nicotiana benthamiana* is a Solanaceae plant with exceptional susceptibility to virus infection, and the main experimental species for the development RVTs. One such technology is virus-induced gene silencing (VIGS), which leverages engineered viral vectors for the targeted downregulation of endogenous genes or gene families for their functional characterization [37, 38].

Transient reprogramming of plant traits is achievable through RVTs [12]. By virus-mediated overexpression of key biosynthetic enzymes or transcription factors, carotenoid and anthocyanin metabolism and storage can be rewired to enable biofortification of edible parts [12, 39]. In a transgenic *N. benthamiana* line carrying a catalytic dead Cas9 fused to autonomous transcriptional activation domains, virus-mediated delivery of guide RNA (gRNA) resulted in strong activation of targeted gene loci, allowing programmable transcriptional regulation and specific alterations of the plant metabolism [40].

Precise and heritable modifications of plant genomic sequences are possible through virus-induced gene editing (VIGE), which relies on engineered viruses to deliver CRISPR–Cas components into plant cells [41]. By using a transgenic line of *N. benthamiana* constitutively expressing Cas9, RNA virus-mediated expression of gRNA incorporating cell-to-cell mobility elements allowed to recover progeny with mutations at the targeted loci with frequencies of 60% to 100% [42].

Advances in reverse genetics of negative-stranded RNA viruses, including those of the families *Rhabdoviridae* and *Tospoviridae*, resulted in viral vectors with unprecedented cargo capacity and stability. These enable to deliver to plant cells large CRISPR–Cas cassettes (~4.5 kb) for simultaneous expression of gRNA alongside Cas9 or Cas12 nucleases, and their engineered base editor versions [13, 43, 44]. Overcoming the need for transgenic Cas-expressing lines in VIGE strategies, RVTs based on barley yellow striate mosaic virus (BYSMV), sonchus yellow net virus (SYNV), and tomato spotted wilt virus (TSWV) allow gene and base editing in non-transgenic hosts.

Viral synthetic genomics is a mature field. Improved T-DNA binary vectors [45] and the rationally designed SynViP [46], a synthetic genomics framework with plant virome capacity, allow high-throughput biological characterization [47] and engineering of plant viruses for applications including VIGS and VIGE [14, 48]. JoinTRV, a one-*Agrobacterium*/two-vector approach, was recently conceived for simultaneous inoculation of the two genomic components of an RNA virus to enable robust VIGS, using low *Agrobacterium* amounts, and heritable VIGE [48]. Given its simplified logistics, the JoinTRV concept could be an attractive option for industrial applications using multipartite viral vectors in general.

### Deploying viruses to improve Solanaceae crop traits

Advances in RVTs developed in *N. benthamiana* have been successfully transferred to main and underutilized solanaceous crops for their functional genomics [49, 50]. Given its robustness and ease of applicability, VIGS has contributed to elucidate the genetics of fruit development and ripening in tomato [38, 51], plant immune responses in potato and pea eggplant (*Solanum torvum*) [52, 53], specialized metabolite biosynthesis in eggplant, peppers, black nightshade (*Solanum nigrum*), lulo (*S. quitoense*), *Physalis* spp., and goji (*Lycium* spp.) [54–60], among others.

RVTs allow the transient reprogramming of agronomic traits in tomato and pepper. Early flowering for speed breeding was achieved through RNA virus-mediated overexpression of *FLOWERING LOCUS T* (*FT*) orthologues [12, 61]. Further, viral delivery of key enzymes and transcription factors resulted in improved traits such as drought stress tolerance, dwarfism, and anthocyanin accumulation for fruit biofortification [12].

Tomatoes with high levels of pigments were obtained by using geminiviral replicons for local delivery of CRISPR–Cas and donor cassettes, which enabled the site specific integration of an heterologous promoter sequence upstream an anthocyanin biosynthetic regulator [62]. Gene and base editing events of *PHYTOENE DESATURASE* (*PDS*)—an endogenous reporter gene whose loss-of-function mutants show an albino phenotype—were achieved through VIGE in tomato [13–15], as well as in sweet, habanero, and tabasco peppers (*C. annuum, Capsicum chinense*, and *Capsicum frutescens*) [13], and, more recently, in potato and eggplant [15]. In a Cas9 tomato line, virus-mediated expression of a repair exonuclease and gRNA led to multinucleotide deletions for functional disruption of noncoding DNA sequences [63], including cis-acting regulatory elements and microRNA genes. Fruit coloration is a multigenic trait with importance for fresh market tomatoes. Breeding of fruit color was reported by VIGE of the tomato *STAYGREEN 1* (*SGR1*), a gene involved in the chlorophyll catabolism during fruit ripening. Mutant progeny displayed a *green-flesh* phenotype characterized by recolored brown fruits with high chlorophyll levels [14], providing the first example of VIGE for agronomic trait breeding in the Solanaceae.

Strategies based on RVTs may enhance performance of underutilized crop, delivering immediate benefits to local food systems. For instance, the internode length in tomato and groundcherry is regulated by *ER* orthologues [29], making them attractive targets for enhancing the growth habit of underutilized crops (Table 1). Targeted *ER* mutagenesis was recently achieved by VIGE in the ornamental Chinese lantern (*Physalis alkekengi*) [13], a relative of groundcherry and tomatillo (*Physalis* spp.), underscoring the large potential of RVTs for the genetic improvement of local food crops.

RVTs may offer a way to increase the mechanization and nutritional properties of Solanaceae plants (Table 1). For instance, inactivation of *FS8.1*, a GT-2-like transcription factor, results in crush-resistant tomatoes suitable to machine harvesting [33], whereas mutagenesis of the key biosynthetic enzyme *STEROL SIDE CHAIN REDUCTASE 2* (*SSR2*) or the transcription factor *GLYCOALKALOID METABOLISM 9* (*GAME9*) has yielded tomato and potato plants with reduced levels of toxic steroidal glycoalkaloids, without compromising plant growth [30,31]. Additionally, *7-DEHYDROCHOLESTEROL REDUCTASE 2* (7-DR2) inactivation rerouted the steroidal glycoalkaloid biosynthesis to yield tomatoes with low antinutritional activity and high content of provitamin D_3_, acting as a human diet supplement against vitamin D deficiencies [32].

### Harnessing the virosphere diversity for biotechnology upgrade

Many RVTs were developed and functionally tweaked in *N. benthamiana* [37]; their applicability to crops may be facilitated by a portfolio of RVTs based on broad-host range or divergent viruses. For example, a TSWV vector system was successfully applied in a variety of crops, including tobacco, tomato, peppers (*Capsicum* spp.), peanut, as well as ornamentals (*P. alkekengi*) [13]. Whilst PVX (genus *Potexvirus*)—repurposed for VIGE—showed infectivity in a modern tomato with introgressed resistances that preclude the use of systems based on tospoviruses, tobamoviruses, and geminiviruses [14].

Local detection of 125 viruses, including 79 novel species, was reported through viromics characterization of tomato agroecosystems in a nation-wide scale [64]. Repurposing local viruses from the pool of >10 000 plant viruses awaiting characterization [65] may help overcome legislative barriers that constrain the use of RVTs locally classified as quarantine pathogens. For instance, tobacco rattle virus (TRV; *Tobravirus*) is widely used in VIGS and VIGE, but its use is controlled in certain countries. To overcome the TRV regulatory restriction in Brazil, a new viral vector system was developed using a local tobravirus, pepper ringspot virus (PepRSV) [66]. Furthermore, exploring viral taxa beyond the mainstream may provide novel tools and viral systems with superior intrinsic features, such as large cargo capacity and stability, as recently reported for tomato chlorosis virus (ToCV; *Crinivirus*) and eggplant mottled dwarf virus (EMDV; *Alphanucleorhabdovirus*) [67, 68].

## Conclusions

Farmed crops and livestock show a non-random phylogenetic distribution within the global biodiversity [2]. Here, we have detected a significant overrepresentation of the Solanaceae in terms of available genomic resources, species used for human nutrition, as well as in its contribution to understanding the virosphere diversity.

RVTs offer high-yield tools for delivering exogenous sequences into plant cells, a feature that has been extensively utilized in both basic and applied research applications [10]. The ease of design and rapid prototyping of engineered virus systems contribute to enhanced flexibility and reduced costs of technology development.

Crops of the Solanaceae have benefited from the widespread adoption of *N. benthamiana* as a model host for plant virology studies and RVTs development. This is exemplified by the successful RVTs implementation in tomato for functional genomics research and, more recently, for the transient and heritable reprogramming of agronomic traits [12–15]. Lesser-known species within the family also serve as local food sources. By leveraging the available genomic resources and knowledge gained from model crops, we have highlighted here the potential of RVTs for functional genomics and genetic improvement of underutilized solanaceous crops, overall contributing to enhancing local and global human nutrition.

Finally, plant viruses are being commercialized as cross-protectant and herbicidal biologicals [35, 36]. Although no engineered virus has been yet registered for agricultural use, multiple RVTs have gained societal acceptance with proven benefits for human and veterinary health [11]. Considering these factors alongside the envisioned applications of engineered plant viruses for crop reprogramming [11], we foresee a pivotal role of RVTs in shaping the evolving horizons of agricultural innovation, fostering sustainable and resilient food production systems.

## Methods

### Data sources

Global crop production values (year 2022) were from FAOSTAT (December 2023 update, https://www.fao.org/faostat/en/#data/QCL). List of plant species cultivated for food were from the Crop_Origins_Phylo_v_live dataset of Crop Origins (https://github.com/rubenmilla/Crop_Origins_Phylo) [69]; information on species richness per plant family [70], and on wild and cultivated Solanaceae edible species was reported [7]. Numbers of assembled genomes per plant family were retrieved from a December 2023 update [71], and plant phylogenetic relationships were from TimeTree (http://www.timetree.org/). Recognized plant virus species were obtained from the International Committee for the Taxonomy of Viruses (ICTV; virus metadata resource VMR_MSL38_v2, https://ictv.global/vmr), and hosts of first discovery were assigned by Virus-Host database [72] and literature searches.

### Statistics

Hypergeometric test (upper cumulative distribution probability) was used to identify plant families where the observed numbers of assembled genomes and reported food crops differed significantly from those expected based on species richness (i.e. the probability of a family having species with available genomic resources or species that have undergone some sort of domestication). Given a list of recognized plant virus species and the hosts of first discovery, a two-sided Grubbs’ test was used to identify significant outliers (i.e. host families whose numbers of assigned plant virus species significantly differ from the rest). Significance levels (*P*) are indicated in the figures and in supplementary data.

## Supplementary Material

Web_Material_uhae205

## Data Availability

Supplementary data accompanies this article. Requests for further information should be directed to and will be fulfilled by the corresponding author.
