# Supplementary material for: Deploying deep Solanaceae domestication and virus biotechnology knowledge to enhance food system performance and diversity
Source: Hortic Res. 2024 Jul 27;11(9):uhae205. doi: 10.1093/hr/uhae205 (PMC11403206; doi:10.1093/hr/uhae205)
Supplement: Web_Material_uhae205 [file web_material_uhae205.pdf]

SUPPLEMENTARY DATA

Deploying deep Solanaceae domestication and virus biotechnology knowledge to enhance food system performance and diversity

Fabio Pasin[1, †, *], Mireia Uranga[2, †], Raghavan Charudattan[3, †], Choon-Tak Kwon[4, †]

[1] Instituto de Biología Molecular y Celular de Plantas (IBMCP), Consejo Superior de Investigaciones Científicas – Universitat Politècnica de València (CSIC-UPV), 46011 Valencia, Spain; email:

[2] Laboratory for Plant Genetics and Crop Improvement, Division of Crop Biotechnics, Department of Biosystems, KU Leuven, 3001 Heverlee, Belgium; KU Leuven Plant Institute (LPI), KU Leuven, 3001 Heverlee, Belgium; email: mireia.urangaruizdeeguino@kuleuven.be

[3] Emeritus Professor, Plant Pathology Department, University of Florida, 32609 Gainesville, FL, USA; BioProdex, Inc., 32609 Gainesville, FL, USA; email: rcharudattan@bioprodex.com

[4] Graduate School of Green-Bio Science, Kyung Hee University, 17104 Yongin, Republic of Korea; Department of Smart Farm Science, Kyung Hee University, 17104 Yongin, Republic of Korea; email:

[†] **ORCID:** F.P., 0000-0002-9620-4301; M.U., 0000-0001-6343-4031; R.C., 0000-0003-2148-3286; C.-T.K., 0000-0002-3589-1662

*** Correspondence:** Fabio Pasin, IBMCP (CSIC-UPV), Ingeniero Fausto Elio, s/n, 46011 Valencia, Spain; Tel.: +34 963877893; Fax. +34 963877859; email: f.pasin@csic.es

CONTENTS

Table S1. Cumulative world crop production values per plant family
Table S2. Representation of crop families with assembled genomes
Table S3. Representation of plant families with species cultivated for human food
Table S4. Available genomic and transcriptomic resources of the Solanaceae
Table S5. Viral genera whose representative species were first discovered in Solanaceae hosts
Table S6. Cumulative numbers of viral species per plant family of first discovery

Table S1. Cumulative world crop production values per plant family

Plant family[1]	World production[2] (tonnes)
Poaceae	4988928875
**Solanaceae**	662080151
Fabaceae	546445133
Arecaceae	499286533
Euphorbiaceae	330408754
Amaranthaceae	294273887
Cucurbitaceae	247009073
Brassicaceae	186736011
Rosaceae	183978994
Musaceae	179263140
Rutaceae	166303421
Amaryllidaceae	146845388
Dioscoreaceae	88257159
Malvaceae	87373693
Convolvulaceae	86410355
Asteraceae	84048079
Vitaceae	74942573
Apiaceae	44984360
Theaceae	29760668
Bromeliaceae	29361138
Oleaceae	21449868
Araceae	18114536
Caricaceae	13822328
Rubiaceae	10782334
Lauraceae	9200799
Asparagaceae	8824150
Pedaliaceae	6741479
Anacardiaceae	6209533
Zingiberaceae	4874216
Actinidiaceae	4539471
Ebenaceae	4436475
Linaceae	3973932
Juglandaceae	3874025
Ericaceae	3597662
Polygonaceae	2235193
Fagaceae	2131241
Aquifoliaceae	1653167
Moraceae	1242449
Betulaceae	1195732
Sapotaceae	815825
Piperaceae	812674
Cannabaceae	200812
Myrtaceae	183452
Myristicaceae	138888
Grossulariaceae	95425
Lecythidaceae	79278
Lamiaceae	51081
Orchidaceae	7704

Basellaceae	n.a.
Talinaceae	n.a.
Cactaceae	n.a.
Clusiaceae	n.a.
Elaeagnaceae	n.a.
Passifloraceae	n.a.
Muntingiaceae	n.a.
Nelumbonaceae	n.a.
Cabombaceae	n.a.
Saururaceae	n.a.
Cannaceae	n.a.
Hydroleaceae	n.a.
Tropaeolaceae	n.a.
Irvingiaceae	n.a.
Moringaceae	n.a.
Martyniaceae	n.a.
Bixaceae	n.a.
Annonaceae	n.a.
Caryocaraceae	n.a.
Sapindaceae	n.a.
Phytolaccaceae	n.a.
Oxalidaceae	n.a.
Burseraceae	n.a.
Typhaceae	n.a.
Myricaceae	n.a.
Nymphaeaceae	n.a.
Liliaceae	n.a.
Schisandraceae	n.a.
Portulacaceae	n.a.
Alismataceae	n.a.
Phyllanthaceae	n.a.
Achariaceae	n.a.
Malpighiaceae	n.a.
Calophyllaceae	n.a.
Adoxaceae	n.a.
Montiaceae	n.a.
Bignoniaceae	n.a.
Marantaceae	n.a.
Alstroemeriaceae	n.a.
Salicaceae	n.a.
Meliaceae	n.a.
Lythraceae	n.a.
Zygophyllaceae	n.a.
Pandanaceae	n.a.
Capparaceae	n.a.
Caprifoliaceae	n.a.
Rhamnaceae	n.a.
Combretaceae	n.a.
Chrysobalanaceae	n.a.
Elaeocarpaceae	n.a.
Asphodelaceae	n.a.

Polygalaceae	n.a.
Verbenaceae	n.a.
Araliaceae	n.a.
Plantaginaceae	n.a.
Celastraceae	n.a.
Crassulaceae	n.a.
Proteaceae	n.a.
Boraginaceae	n.a.
Aizoaceae	n.a.
Iridaceae	n.a.
Campanulaceae	n.a.
Ranunculaceae	n.a.
Cyperaceae	n.a.
Apocynaceae	n.a.

[1] Families with species cultivated for food (Crop Origins, https://github.com/rubenmilla/Crop_Origins_Phylo)

[2] Data of year 2022 (FAOSTAT, https://www.fao.org/faostat/en/#data/QCL); n.a., not available.

Table S2. Representation of crop families with assembled genomes

Plant family[1]	Total species[2]	Assembled genomes[3] (species)	*P*	-log10(*P*)	Overrepresentation
Brassicaceae	3628	112	5.62E-50	49.25	TRUE
**Solanaceae**	2600	80	8.31E-36	35.08	TRUE
Rosaceae	2950	77	6.77E-30	29.17	TRUE
Juglandaceae	50	17	1.33E-26	25.88	TRUE
Poaceae	12000	132	2.76E-15	14.56	TRUE
Oleaceae	790	28	6.91E-15	14.16	TRUE
Musaceae	91	11	2.62E-12	11.58	TRUE
Vitaceae	910	26	7.83E-12	11.11	TRUE
Betulaceae	167	13	8.17E-12	11.09	TRUE
Salicaceae	1220	28	1.89E-10	9.72	TRUE
Cucurbitaceae	965	23	3.83E-09	8.42	TRUE
Pedaliaceae	75	8	7.05E-09	8.15	TRUE
Fagaceae	927	18	3.29E-06	5.48	TRUE
Rutaceae	2070	26	5.99E-05	4.22	TRUE
Moraceae	1180	18	7.69E-05	4.11	TRUE
Nelumbonaceae	3	2	8.26E-05	4.08	TRUE
Lythraceae	620	12	1.40E-04	3.85	TRUE
Cabombaceae	6	2	4.08E-04	3.39	TRUE
Actinidiaceae	360	8	7.30E-04	3.14	TRUE
Cannaceae	10	2	1.21E-03	2.92	TRUE
Cannabaceae	100	4	2.00E-03	2.70	TRUE
Malvaceae	4225	37	2.24E-03	2.65	TRUE
Typhaceae	51	3	2.50E-03	2.60	TRUE
Elaeagnaceae	60	3	3.97E-03	2.40	TRUE
Meliaceae	600	9	5.03E-03	2.30	TRUE
Nymphaeaceae	70	3	6.11E-03	2.21	TRUE
Sapindaceae	1860	17	2.18E-02	1.66	TRUE
Saururaceae	6	1	3.11E-02	1.51	TRUE
Myrtaceae	5950	42	3.62E-02	1.44	TRUE
Theaceae	240	4	3.89E-02	1.41	TRUE
Moringaceae	13	1	6.62E-02	1.18	FALSE
Polygonaceae	1200	10	1.06E-01	0.98	FALSE
Portulacaceae	115	2	1.23E-01	0.91	FALSE
Amaranthaceae	2040	15	1.25E-01	0.90	FALSE
Caryocaraceae	26	1	1.28E-01	0.89	FALSE
Combretaceae	530	5	1.49E-01	0.83	FALSE
Linaceae	255	3	1.52E-01	0.82	FALSE
Phytolaccaceae	33	1	1.60E-01	0.80	FALSE
Caricaceae	35	1	1.68E-01	0.77	FALSE
Anacardiaceae	860	7	1.71E-01	0.77	FALSE
Myricaceae	57	1	2.60E-01	0.59	FALSE
Dioscoreaceae	715	5	3.24E-01	0.49	FALSE
Zingiberaceae	1600	10	3.35E-01	0.47	FALSE
Burseraceae	615	4	4.05E-01	0.39	FALSE
Ericaceae	4250	23	4.73E-01	0.33	FALSE
Aquifoliaceae	500	3	4.89E-01	0.31	FALSE
Plantaginaceae	1900	10	5.41E-01	0.27	FALSE
Lecythidaceae	355	2	5.57E-01	0.25	FALSE

Fabaceae	19500	101	5.77E-01	0.24	FALSE
Convolvulaceae	1660	8	6.44E-01	0.19	FALSE
Bignoniaceae	870	4	6.71E-01	0.17	FALSE
Passifloraceae	980	4	7.57E-01	0.12	FALSE
Araliaceae	1650	7	7.64E-01	0.12	FALSE
Proteaceae	1660	7	7.69E-01	0.11	FALSE
Zygophyllaceae	285	1	7.78E-01	0.11	FALSE
Ebenaceae	800	3	7.92E-01	0.10	FALSE
Caprifoliaceae	825	3	8.09E-01	0.09	FALSE
Capparaceae	324	1	8.19E-01	0.09	FALSE
Lamiaceae	7530	34	8.38E-01	0.08	FALSE
Asphodelaceae	900	3	8.52E-01	0.07	FALSE
Arecaceae	2600	10	8.76E-01	0.06	FALSE
Rhamnaceae	950	3	8.76E-01	0.06	FALSE
Cactaceae	1750	6	8.97E-01	0.05	FALSE
Calophyllaceae	475	1	9.18E-01	0.04	FALSE
Crassulaceae	1400	4	9.36E-01	0.03	FALSE
Oxalidaceae	570	1	9.51E-01	0.02	FALSE
Marantaceae	570	1	9.51E-01	0.02	FALSE
Elaeocarpaceae	615	1	9.61E-01	0.02	FALSE
Sapotaceae	1273	3	9.63E-01	0.02	FALSE
Amaryllidaceae	1600	4	9.69E-01	0.01	FALSE
Apiaceae	3575	11	9.81E-01	0.01	FALSE
Polygalaceae	900	1	9.91E-01	0.00	FALSE
Lauraceae	2850	7	9.93E-01	0.00	FALSE
Celastraceae	1350	2	9.93E-01	0.00	FALSE
Ranunculaceae	2346	5	9.94E-01	0.00	FALSE
Araceae	3750	10	9.94E-01	0.00	FALSE
Verbenaceae	1000	1	9.95E-01	0.00	FALSE
Campanulaceae	2300	3	1.00E+00	0.00	FALSE
Phyllanthaceae	2050	2	1.00E+00	0.00	FALSE
Annonaceae	2500	3	1.00E+00	0.00	FALSE
Apocynaceae	5100	11	1.00E+00	0.00	FALSE
Asparagaceae	2900	4	1.00E+00	0.00	FALSE
Aizoaceae	1900	1	1.00E+00	0.00	FALSE
Cyperaceae	5500	11	1.00E+00	0.00	FALSE
Boraginaceae	2535	2	1.00E+00	0.00	FALSE
Bromeliaceae	3475	4	1.00E+00	0.00	FALSE
Iridaceae	2244	1	1.00E+00	0.00	FALSE
Euphorbiaceae	6252	10	1.00E+00	0.00	FALSE
Piperaceae	3700	1	1.00E+00	0.00	FALSE
Rubiaceae	13620	17	1.00E+00	0.00	FALSE
Asteraceae	24700	36	1.00E+00	0.00	FALSE
Myristicaceae	520	0	1.00E+00	0.00	FALSE
Grossulariaceae	150	0	1.00E+00	0.00	FALSE
Orchidaceae	28000	20	1.00E+00	0.00	FALSE
Basellaceae	19	0	1.00E+00	0.00	FALSE
Talinaceae	28	0	1.00E+00	0.00	FALSE
Clusiaceae	750	0	1.00E+00	0.00	FALSE
Muntingiaceae	3	0	1.00E+00	0.00	FALSE
Hydroleaceae	12	0	1.00E+00	0.00	FALSE

Tropaeolaceae	94	0	1.00E+00	0.00	FALSE
Irvingiaceae	13	0	1.00E+00	0.00	FALSE
Martyniaceae	16	0	1.00E+00	0.00	FALSE
Bixaceae	23	0	1.00E+00	0.00	FALSE
Liliaceae	705	0	1.00E+00	0.00	FALSE
Schisandraceae	85	0	1.00E+00	0.00	FALSE
Alismataceae	115	0	1.00E+00	0.00	FALSE
Achariaceae	155	0	1.00E+00	0.00	FALSE
Malpighiaceae	1315	0	1.00E+00	0.00	FALSE
Adoxaceae	225	0	1.00E+00	0.00	FALSE
Montiaceae	230	0	1.00E+00	0.00	FALSE
Alstroemeriaceae	254	0	1.00E+00	0.00	FALSE
Pandanaceae	982	0	1.00E+00	0.00	FALSE
Chrysobalanacea	533	0	1.00E+00	0.00	FALSE

[1] Families with species cultivated for food (Crop Origins, https://github.com/rubenmilla/Crop_Origins_Phylo)

[2] Numbers from Christenhusz & Byng, Phytotaxa 2016; 261: 201.

[3] Numbers from Xie et al., Nat Plants 2024; 10: 551–566.

Table S3. Representation of plant families with species cultivated for human food

Plant family[1]	Total species[2]	Food crop species[1]	*P*	-log10(*P*)	Overrepresentation
Rosaceae	2950	54	2.05E-23	22.69	TRUE
Juglandaceae	50	8	7.04E-12	11.15	TRUE
**Solanaceae**	2600	33	1.49E-10	9.83	TRUE
Cucurbitaceae	965	20	2.04E-10	9.69	TRUE
Grossulariaceae	150	8	5.14E-08	7.29	TRUE
Dioscoreaceae	715	14	2.05E-07	6.69	TRUE
Anacardiaceae	860	15	3.28E-07	6.48	TRUE
Basellaceae	19	4	4.58E-07	6.34	TRUE
Amaranthaceae	2040	22	2.41E-06	5.62	TRUE
Zingiberaceae	1600	19	3.00E-06	5.52	TRUE
Talinaceae	28	3	1.14E-04	3.94	TRUE
Pedaliaceae	75	4	1.24E-04	3.91	TRUE
Moraceae	1180	13	2.18E-04	3.66	TRUE
Caricaceae	35	3	2.24E-04	3.65	TRUE
Betulaceae	167	5	2.67E-04	3.57	TRUE
Rutaceae	2070	18	2.79E-04	3.55	TRUE
Cactaceae	1750	16	3.49E-04	3.46	TRUE
Clusiaceae	750	9	1.09E-03	2.96	TRUE
Elaeagnaceae	60	3	1.10E-03	2.96	TRUE
Sapotaceae	1273	12	1.42E-03	2.85	TRUE
Passifloraceae	980	10	1.96E-03	2.71	TRUE
Musaceae	91	3	3.62E-03	2.44	TRUE
Apiaceae	3575	22	5.26E-03	2.28	TRUE
Actinidiaceae	360	5	7.60E-03	2.12	TRUE
Polygonaceae	1200	10	7.91E-03	2.10	TRUE
Muntingiaceae	3	1	9.98E-03	2.00	TRUE
Nelumbonaceae	3	1	9.98E-03	2.00	TRUE
Malvaceae	4225	23	1.69E-02	1.77	TRUE
Ebenaceae	800	7	1.91E-02	1.72	TRUE
Cabombaceae	6	1	1.99E-02	1.70	TRUE
Saururaceae	6	1	1.99E-02	1.70	TRUE
Amaryllidaceae	1600	11	2.04E-02	1.69	TRUE
Cannaceae	10	1	3.29E-02	1.48	TRUE
Brassicaceae	3628	19	3.87E-02	1.41	TRUE
Hydroleaceae	12	1	3.93E-02	1.41	TRUE
Tropaeolaceae	94	2	3.97E-02	1.40	TRUE
Irvingiaceae	13	1	4.25E-02	1.37	TRUE
Moringaceae	13	1	4.25E-02	1.37	TRUE
Martyniaceae	16	1	5.21E-02	1.28	FALSE
Bixaceae	23	1	7.40E-02	1.13	FALSE
Annonaceae	2500	13	8.03E-02	1.10	FALSE
Caryocaraceae	26	1	8.32E-02	1.08	FALSE
Sapindaceae	1860	10	9.77E-02	1.01	FALSE
Phytolaccaceae	33	1	1.04E-01	0.98	FALSE
Lecythidaceae	355	3	1.17E-01	0.93	FALSE
Oxalidaceae	570	4	1.25E-01	0.90	FALSE
Fabaceae	19500	74	1.38E-01	0.86	FALSE
Burseraceae	615	4	1.52E-01	0.82	FALSE
Typhaceae	51	1	1.57E-01	0.80	FALSE
Arecaceae	2600	12	1.65E-01	0.78	FALSE
Myricaceae	57	1	1.73E-01	0.76	FALSE
Theaceae	240	2	1.91E-01	0.72	FALSE
Fagaceae	927	5	2.00E-01	0.70	FALSE
Nymphaeaceae	70	1	2.09E-01	0.68	FALSE
Liliaceae	705	4	2.11E-01	0.68	FALSE
Schisandraceae	85	1	2.47E-01	0.61	FALSE
Araceae	3750	15	2.75E-01	0.56	FALSE
Cannabaceae	100	1	2.84E-01	0.55	FALSE
Alismataceae	115	1	3.19E-01	0.50	FALSE
Portulacaceae	115	1	3.19E-01	0.50	FALSE
Poaceae	12000	43	3.37E-01	0.47	FALSE
Phyllanthaceae	2050	8	3.77E-01	0.42	FALSE
Achariaceae	155	1	4.04E-01	0.39	FALSE
Myrtaceae	5950	21	4.28E-01	0.37	FALSE
Malpighiaceae	1315	5	4.47E-01	0.35	FALSE

Calophyllaceae	475	2	4.71E-01	0.33	FALSE
Lauraceae	2850	10	4.80E-01	0.32	FALSE
Oleaceae	790	3	4.91E-01	0.31	FALSE
Adoxaceae	225	1	5.29E-01	0.28	FALSE
Montiaceae	230	1	5.37E-01	0.27	FALSE
Bignoniaceae	870	3	5.55E-01	0.26	FALSE
Marantaceae	570	2	5.68E-01	0.25	FALSE
Alstroemeriaceae	254	1	5.72E-01	0.24	FALSE
Linaceae	255	1	5.74E-01	0.24	FALSE
Salicaceae	1220	4	5.81E-01	0.24	FALSE
Vitaceae	910	3	5.86E-01	0.23	FALSE
Meliaceae	600	2	5.95E-01	0.23	FALSE
Lythraceae	620	2	6.13E-01	0.21	FALSE
Zygophyllaceae	285	1	6.14E-01	0.21	FALSE
Pandanaceae	982	3	6.37E-01	0.20	FALSE
Convolvulaceae	1660	5	6.50E-01	0.19	FALSE
Capparaceae	324	1	6.62E-01	0.18	FALSE
Caprifoliaceae	825	2	7.62E-01	0.12	FALSE
Aquifoliaceae	500	1	8.12E-01	0.09	FALSE
Myristicaceae	520	1	8.24E-01	0.08	FALSE
Rhamnaceae	950	2	8.26E-01	0.08	FALSE
Combretaceae	530	1	8.30E-01	0.08	FALSE
Chrysobalanacea	533	1	8.32E-01	0.08	FALSE
Elaeocarpaceae	615	1	8.72E-01	0.06	FALSE
Asphodelaceae	900	1	9.51E-01	0.02	FALSE
Polygalaceae	900	1	9.51E-01	0.02	FALSE
Verbenaceae	1000	1	9.65E-01	0.02	FALSE
Araliaceae	1650	2	9.74E-01	0.01	FALSE
Lamiaceae	7530	16	9.81E-01	0.01	FALSE
Piperaceae	3700	6	9.84E-01	0.01	FALSE
Plantaginaceae	1900	2	9.87E-01	0.01	FALSE
Asparagaceae	2900	4	9.87E-01	0.01	FALSE
Celastraceae	1350	1	9.89E-01	0.00	FALSE
Crassulaceae	1400	1	9.91E-01	0.00	FALSE
Proteaceae	1660	1	9.96E-01	0.00	FALSE
Boraginaceae	2535	2	9.98E-01	0.00	FALSE
Aizoaceae	1900	1	9.98E-01	0.00	FALSE
Bromeliaceae	3475	3	9.99E-01	0.00	FALSE
Iridaceae	2244	1	9.99E-01	0.00	FALSE
Campanulaceae	2300	1	1.00E+00	0.00	FALSE
Ranunculaceae	2346	1	1.00E+00	0.00	FALSE
Euphorbiaceae	6252	8	1.00E+00	0.00	FALSE
Ericaceae	4250	4	1.00E+00	0.00	FALSE
Cyperaceae	5500	3	1.00E+00	0.00	FALSE
Apocynaceae	5100	2	1.00E+00	0.00	FALSE
Rubiaceae	13620	7	1.00E+00	0.00	FALSE
Asteraceae	24700	25	1.00E+00	0.00	FALSE
Orchidaceae	28000	3	1.00E+00	0.00	FALSE

[1] Families with species cultivated for food, and number of crop species per family (Crop Origins, https://github.com/rubenmilla/Crop_Origins_Phylo)

[2] Numbers from Christenhusz & Byng, Phytotaxa 2016; 261: 201.

Table S4. Available genomic and transcriptomic resources of the Solanaceae

Species	Resources[1]	Publication	DOI	NCBI data accession numbers/links
Alkekengi officinarum var. franchetii	Transcriptome	Plant Communications	10.1016/j.xplc.2023.100595	DRR048294
Anisodus acutangulus	Genome	Nature Plants	10.1038/s41477-024-01655-6	http://ibi.zju.edu.cn/N3database/index.php
Anisodus luridus	Genome	Cell	10.1016/j.cell.2023.04.008	PRJNA839598
Anisodus stramoniifolius	Transcriptome	Plant Communications	10.1016/j.xplc.2023.100595	PRJNA827705
Archiphysalis chamaesarachoides	Transcriptome	Plant Communications	10.1016/j.xplc.2023.100595	PRJNA827705
Athenaea wettsteiniana	Genome	Plant Communications	10.1016/j.xplc.2023.100595	PRJNA827705
Atropa belladonna	Genome	Nature Plants	10.1038/s41477-024-01655-6	http://ibi.zju.edu.cn/N3database/index.php
Atropa belladonna	Transcriptome	Plant Communications	10.1016/j.xplc.2023.100595	ERR2040625
Browallia americana	Transcriptome	Plant Communications	10.1016/j.xplc.2023.100595	PRJNA827705
Brugmansia aurea	Transcriptome	Plant Communications	10.1016/j.xplc.2023.100595	PRJNA827705
Brugmansia sanguinea	Transcriptome	Plant Communications	10.1016/j.xplc.2023.100595	ERR2040635
Brunfelsia americana	Transcriptome	Plant Communications	10.1016/j.xplc.2023.100595	PRJNA827705
Brunfelsia grandiflora	Transcriptome	Plant Communications	10.1016/j.xplc.2023.100595	PRJNA827705
Calibrachoa hybrids	Transcriptome	Plant Communications	10.1016/j.xplc.2023.100595	PRJNA827705
Capsicum annuum	Genome	Nature Plants	10.1038/s41477-024-01655-6	http://ibi.zju.edu.cn/N3database/index.php
Capsicum baccatum	Genome	Nature Plants	10.1038/s41477-024-01655-6	http://ibi.zju.edu.cn/N3database/index.php
Capsicum chinense	Genome	Nature Plants	10.1038/s41477-024-01655-6	http://ibi.zju.edu.cn/N3database/index.php
Capsicum pubescens	Genome	Nature Plants	10.1038/s41477-024-01655-6	http://ibi.zju.edu.cn/N3database/index.php
Cestrum capsulare	Genome	Plant Communications	10.1016/j.xplc.2023.100595	PRJNA827705
Cestrum diurnum	Transcriptome	Plant Communications	10.1016/j.xplc.2023.100595	PRJNA827705
Cestrum parqui	Transcriptome	Plant Communications	10.1016/j.xplc.2023.100595	PRJNA827705
Cuatresia colombiana	Genome	Plant Communications	10.1016/j.xplc.2023.100595	PRJNA827705
Datura inoxia	Genome	Cell	10.1016/j.cell.2023.04.008	PRJNA839598
Datura stramonium	Genome	Cell	10.1016/j.cell.2023.04.008	PRJNA839598
Datura wrightii	Transcriptome	Plant Communications	10.1016/j.xplc.2023.100595	PRJNA827705
Datura stramonium	Transcriptome	Plant Communications	10.1016/j.xplc.2023.100595	PRJNA827705
Dunalia spinosa	Transcriptome	Plant Communications	10.1016/j.xplc.2023.100595	PRJNA827705
Goetzea ekmanii	Transcriptome	Plant Communications	10.1016/j.xplc.2023.100595	PRJNA827705
Grammosolen truncatus	Genome	Plant Communications	10.1016/j.xplc.2023.100595	PRJNA827705
Hyoscyamus niger	Transcriptome	Plant Communications	10.1016/j.xplc.2023.100595	SRR2231029
Iochroma arborescens	Transcriptome	Plant Communications	10.1016/j.xplc.2023.100595	PRJNA827705
Iochroma cyaneum	Genome	Nature Plants	10.1038/s41477-024-01655-6	http://ibi.zju.edu.cn/N3database/index.php
Iochroma cyaneum	Transcriptome	Plant Communications	10.1016/j.xplc.2023.100595	PRJNA827705
Jaborosa laciniata	Transcriptome	Plant Communications	10.1016/j.xplc.2023.100595	PRJNA827705
Jaltomata aijana	Transcriptome	Plant Communications	10.1016/j.xplc.2023.100595	SRR5380899
Jaltomata auriculata	Transcriptome	Plant Communications	10.1016/j.xplc.2023.100595	SRR5380907
Jaltomata biflora	Transcriptome	Plant Communications	10.1016/j.xplc.2023.100595	SRR5380903
Jaltomata calliantha	Transcriptome	Plant Communications	10.1016/j.xplc.2023.100595	SRR5380911
Jaltomata darcyana	Transcriptome	Plant Communications	10.1016/j.xplc.2023.100595	SRR5380913
Jaltomata dendroidea	Transcriptome	Plant Communications	10.1016/j.xplc.2023.100595	SRR5380895
Jaltomata grandibaccata	Transcriptome	Plant Communications	10.1016/j.xplc.2023.100595	SRR5380892
Jaltomata incahuasina	Transcriptome	Plant Communications	10.1016/j.xplc.2023.100595	SRR5380893
Jaltomata procumbens	Transcriptome	Plant Communications	10.1016/j.xplc.2023.100595	SRR5380915
Jaltomata quipuscoae	Transcriptome	Plant Communications	10.1016/j.xplc.2023.100595	SRR5380909
Jaltomata repandidentata	Transcriptome	Plant Communications	10.1016/j.xplc.2023.100595	SRR5380917
Jaltomata sinuosa	Genome	Nature Plants	10.1038/s41477-024-01655-6	http://ibi.zju.edu.cn/N3database/index.php
Jaltomata umbellata	Transcriptome	Plant Communications	10.1016/j.xplc.2023.100595	SRR5380897
Jaltomata yungayensis	Transcriptome	Plant Communications	10.1016/j.xplc.2023.100595	SRR5380905
Juanulloa mexicana	Transcriptome	Plant Communications	10.1016/j.xplc.2023.100595	PRJNA827705
Latua pubiflora	Transcriptome	Plant Communications	10.1016/j.xplc.2023.100595	PRJNA827705
Leucophysalis nana	Transcriptome	Plant Communications	10.1016/j.xplc.2023.100595	PRJNA827705
Lycianthes asarifolia	Transcriptome	New Phytologist	10.1111/nph.19849	PRJNA1078428
Lycianthes biflora	Genome	Cell	10.1016/j.cell.2023.04.008	PRJNA839598
Lycianthes cuchumatanensis	Transcriptome	Plant Communications	10.1016/j.xplc.2023.100595	PRJNA827705
Lycianthes rantonnetii	Transcriptome	Plant Communications	10.1016/j.xplc.2023.100595	PRJNA827705
Lycianthes tricolor	Transcriptome	Plant Communications	10.1016/j.xplc.2023.100595	PRJNA827705
Lycium barbarum	Genome	Cell	10.1016/j.cell.2023.04.008	PRJNA839598
Lycium boerhaviifolium1	Transcriptome	Plant Communications	10.1016/j.xplc.2023.100595	PRJNA827705
Lycium boerhaviifolium2	Transcriptome	Plant Communications	10.1016/j.xplc.2023.100595	PRJNA827705
Lycium ruthenicum	Genome	Nature Plants	10.1038/s41477-024-01655-6	http://ibi.zju.edu.cn/N3database/index.php
Lycium ruthenicum	Transcriptome	Plant Communications	10.1016/j.xplc.2023.100595	SRR3198233
Mandragora caulescens	Transcriptome	Plant Communications	10.1016/j.xplc.2023.100595	PRJNA827705
Mandragora officinarum	Transcriptome	Plant Communications	10.1016/j.xplc.2023.100595	PRJNA827705
Nicandra physalodes	Genome	Cell	10.1016/j.cell.2023.04.008	PRJNA839598
Nicandra physalodes	Transcriptome	Plant Communications	10.1016/j.xplc.2023.100595	PRJNA827705
Nicotiana attenuata	Genome	Nature Plants	10.1038/s41477-024-01655-6	http://ibi.zju.edu.cn/N3database/index.php
Nicotiana attenuata	Transcriptome	Plant Communications	10.1016/j.xplc.2023.100595	PRJNA827705
Nicotiana benthamiana	Genome	Cell	10.1016/j.cell.2023.04.008	PRJNA839598
Nicotiana cordifolia	Transcriptome	Plant Communications	10.1016/j.xplc.2023.100595	SRR2106516
Nicotiana glauca	Genome	Nature Plants	10.1038/s41477-024-01655-6	http://ibi.zju.edu.cn/N3database/index.php
Nicotiana glauca	Transcriptome	Plant Communications	10.1016/j.xplc.2023.100595	SRR2106216
Nicotiana knightiana	Genome	Nature Plants	10.1038/s41477-024-01655-6	http://ibi.zju.edu.cn/N3database/index.php
Nicotiana longiflora	Genome	Cell	10.1016/j.cell.2023.04.008	PRJNA839598
Nicotiana noctiflora	Transcriptome	Plant Communications	10.1016/j.xplc.2023.100595	SRR2106514
Nicotiana obtusifolia	Genome	Nature Plants	10.1038/s41477-024-01655-6	http://ibi.zju.edu.cn/N3database/index.php
Nicotiana otophora	Genome	Nature Plants	10.1038/s41477-024-01655-6	http://ibi.zju.edu.cn/N3database/index.php
Nicotiana paniculata	Genome	Nature Plants	10.1038/s41477-024-01655-6	http://ibi.zju.edu.cn/N3database/index.php
Nicotiana paniculata	Transcriptome	Plant Communications	10.1016/j.xplc.2023.100595	PRJNA827705
Nicotiana rustica	Genome	Nature Plants	10.1038/s41477-024-01655-6	http://ibi.zju.edu.cn/N3database/index.php
Nicotiana setchellii	Transcriptome	Plant Communications	10.1016/j.xplc.2023.100595	SRR2106530
Nicotiana sylvestris	Genome	Nature Plants	10.1038/s41477-024-01655-6	http://ibi.zju.edu.cn/N3database/index.php
Nicotiana tabacum	Genome	Nature Plants	10.1038/s41477-024-01655-6	http://ibi.zju.edu.cn/N3database/index.php
Nicotiana tomentosiformis	Genome	Nature Plants	10.1038/s41477-024-01655-6	http://ibi.zju.edu.cn/N3database/index.php
Nicotiana tomentosiformis	Transcriptome	Plant Communications	10.1016/j.xplc.2023.100595	SRR2106531
Nicotiana undulata	Genome	Nature Plants	10.1038/s41477-024-01655-6	http://ibi.zju.edu.cn/N3database/index.php
Nierembergia linariifolia var. linariifolia	Transcriptome	Plant Communications	10.1016/j.xplc.2023.100595	PRJNA827705
Nolana crassulifolia	Transcriptome	Plant Communications	10.1016/j.xplc.2023.100595	PRJNA827705
Nolana paradoxa	Transcriptome	Plant Communications	10.1016/j.xplc.2023.100595	PRJNA827705
Petunia axillaris	Genome	Nature Plants	10.1038/s41477-024-01655-6	http://ibi.zju.edu.cn/N3database/index.php
Petunia exserta	Transcriptome	Plant Communications	10.1016/j.xplc.2023.100595	PRJNA827705
Petunia inflata	Genome	Nature Plants	10.1038/s41477-024-01655-6	http://ibi.zju.edu.cn/N3database/index.php
Physaliastrum heterophyllum	Transcriptome	Plant Communications	10.1016/j.xplc.2023.100595	PRJNA827705
Physalis angulata	Genome	Cell	10.1016/j.cell.2023.04.008	PRJNA839598
Physalis angulata	Transcriptome	Plant Communications	10.1016/j.xplc.2023.100595	PRJNA827705

Physalis floridana	Genome	Nature Plants	10.1038/s41477-024-01655-6	http://ibi.zju.edu.cn/N3database/index.php
Physalis grisea	Genome	Nature Plants	10.1038/s41477-024-01655-6	http://ibi.zju.edu.cn/N3database/index.php
Physalis longifolia var. subglabrata	Transcriptome	Plant Communications	10.1016/j.xplc.2023.100595	PRJNA827705
Physalis peruviana	Transcriptome	Plant Communications	10.1016/j.xplc.2023.100595	DRR048298
Physalis pruinosa	Genome	Nature Plants	10.1038/s41477-024-01655-6	http://ibi.zju.edu.cn/N3database/index.php
Physochlaina orientalis	Transcriptome	Plant Communications	10.1016/j.xplc.2023.100595	PRJNA827705
Physochlaina praealta	Transcriptome	Plant Communications	10.1016/j.xplc.2023.100595	PRJNA827705
Przewalskia tangutica	Genome	Nature Plants	10.1038/s41477-024-01655-6	http://ibi.zju.edu.cn/N3database/index.php
Przewalskia tangutica	Transcriptome	Plant Communications	10.1016/j.xplc.2023.100595	PRJNA827705
Reyesia chilensis	Transcriptome	Plant Communications	10.1016/j.xplc.2023.100595	PRJNA827705
Reyesia juniperoides	Transcriptome	Plant Communications	10.1016/j.xplc.2023.100595	PRJNA827705
Salpichroa origanifolia	Transcriptome	Plant Communications	10.1016/j.xplc.2023.100595	PRJNA827705
Salpiglossis sinuata	Transcriptome	Plant Communications	10.1016/j.xplc.2023.100595	SRR2229601
Schizanthus litoralis	Transcriptome	Plant Communications	10.1016/j.xplc.2023.100595	PRJNA827705
Schwenckia americana	Transcriptome	Plant Communications	10.1016/j.xplc.2023.100595	PRJNA827705
Sclerophylax spinescens	Genome	Plant Communications	10.1016/j.xplc.2023.100595	PRJNA827705
Solandra grandiflora	Transcriptome	Plant Communications	10.1016/j.xplc.2023.100595	PRJNA827705
Solandra maxima	Transcriptome	Plant Communications	10.1016/j.xplc.2023.100595	PRJNA827705
Solanum abutiloides	Transcriptome	Plant Communications	10.1016/j.xplc.2023.100595	PRJNA827705
Solanum acanthodes	Transcriptome	New Phytologist	10.1111/nph.19849	PRJNA1078428
Solanum acerifolium	Transcriptome	New Phytologist	10.1111/nph.19849	PRJNA1078428
Solanum achorum	Transcriptome	New Phytologist	10.1111/nph.19849	PRJNA1078428
Solanum actaeibotrys	Transcriptome	New Phytologist	10.1111/nph.19849	PRJNA1078428
Solanum aethiopicum	Genome	Nature Plants	10.1038/s41477-024-01655-6	http://ibi.zju.edu.cn/N3database/index.php
Solanum aethiopicum	Transcriptome	Plant Communications	10.1016/j.xplc.2023.100595	SRR2229192
Solanum aff. Velleum	Transcriptome	New Phytologist	10.1111/nph.19849	PRJNA1078428
Solanum americanum	Genome	Cell	10.1016/j.cell.2023.04.008	PRJNA839598
Solanum americanum	Transcriptome	Plant Communications	10.1016/j.xplc.2023.100595	ERR2040626
Solanum anceps	Transcriptome	New Phytologist	10.1111/nph.19849	PRJNA1078428
Solanum andigena	Genome	Nature Plants	10.1038/s41477-024-01655-6	http://ibi.zju.edu.cn/N3database/index.php
Solanum andreanum	Genome	Nature Plants	10.1038/s41477-024-01655-6	http://ibi.zju.edu.cn/N3database/index.php
Solanum aphyodendron	Transcriptome	New Phytologist	10.1111/nph.19849	PRJNA1078428
Solanum appendiculatum	Genome	Nature Plants	10.1038/s41477-024-01655-6	http://ibi.zju.edu.cn/N3database/index.php
Solanum appendiculatum	Transcriptome	New Phytologist	10.1111/nph.19849	PRJNA1078428
Solanum appressum	Transcriptome	New Phytologist	10.1111/nph.19849	PRJNA1078428
Solanum arboreum	Transcriptome	New Phytologist	10.1111/nph.19849	PRJNA1078428
Solanum arcanum	Genome	Nature Plants	10.1038/s41477-024-01655-6	http://ibi.zju.edu.cn/N3database/index.php
Solanum arcanum	Transcriptome	Plant Communications	10.1016/j.xplc.2023.100595	SRR3031976
Solanum argenteum	Transcriptome	New Phytologist	10.1111/nph.19849	PRJNA1078428
Solanum asperolanatum	Transcriptome	New Phytologist	10.1111/nph.19849	PRJNA1078428
Solanum asperum	Transcriptome	New Phytologist	10.1111/nph.19849	PRJNA1078428
Solanum atropurpureum	Genome	Cell	10.1016/j.cell.2023.04.008	PRJNA839598
Solanum atropurpureum	Transcriptome	New Phytologist	10.1111/nph.19849	PRJNA1078428
Solanum aviculare	Transcriptome	New Phytologist	10.1111/nph.19849	PRJNA1078428
Solanum bahamense	Transcriptome	New Phytologist	10.1111/nph.19849	PRJNA1078428
Solanum betaceum	Transcriptome	Plant Communications	10.1016/j.xplc.2023.100595	PRJNA827705
Solanum bicolor	Transcriptome	New Phytologist	10.1111/nph.19849	PRJNA1078428
Solanum bohsiae	Transcriptome	New Phytologist	10.1111/nph.19849	PRJNA1078428
Solanum boliviense	Genome	Nature Plants	10.1038/s41477-024-01655-6	http://ibi.zju.edu.cn/N3database/index.php
Solanum bradei	Transcriptome	New Phytologist	10.1111/nph.19849	PRJNA1078428
Solanum brevicaule	Genome	Nature Plants	10.1038/s41477-024-01655-6	http://ibi.zju.edu.cn/N3database/index.php
Solanum buesii	Genome	Nature Plants	10.1038/s41477-024-01655-6	http://ibi.zju.edu.cn/N3database/index.php
Solanum bukasovii	Genome	Nature Plants	10.1038/s41477-024-01655-6	http://ibi.zju.edu.cn/N3database/index.php
Solanum bulbocastanum	Genome	Nature Plants	10.1038/s41477-024-01655-6	http://ibi.zju.edu.cn/N3database/index.php
Solanum bullatum	Transcriptome	New Phytologist	10.1111/nph.19849	PRJNA1078428
Solanum burkartii	Genome	Nature Plants	10.1038/s41477-024-01655-6	http://ibi.zju.edu.cn/N3database/index.php
Solanum cajamarquense	Genome	Nature Plants	10.1038/s41477-024-01655-6	http://ibi.zju.edu.cn/N3database/index.php
Solanum cajanumense	Transcriptome	New Phytologist	10.1111/nph.19849	PRJNA1078428
Solanum campaniforme	Transcriptome	New Phytologist	10.1111/nph.19849	PRJNA1078428
Solanum candolleanum	Genome	Nature Plants	10.1038/s41477-024-01655-6	http://ibi.zju.edu.cn/N3database/index.php
Solanum capsicoides	Genome	Cell	10.1016/j.cell.2023.04.008	PRJNA839598
Solanum caricaefolium	Transcriptome	New Phytologist	10.1111/nph.19849	PRJNA1078428
Solanum caripense	Transcriptome	Plant Communications	10.1016/j.xplc.2023.100595	SRR2239273
Solanum carolinense	Transcriptome	Plant Communications	10.1016/j.xplc.2023.100595	PRJNA827705
Solanum cassioides	Transcriptome	New Phytologist	10.1111/nph.19849	PRJNA1078428
Solanum castaneum	Transcriptome	New Phytologist	10.1111/nph.19849	PRJNA1078428
Solanum cernuum	Transcriptome	New Phytologist	10.1111/nph.19849	PRJNA1078428
Solanum cf. aspersum	Transcriptome	New Phytologist	10.1111/nph.19849	PRJNA1078428
Solanum cf. lantana	Transcriptome	New Phytologist	10.1111/nph.19849	PRJNA1078428
Solanum cf. melissarum	Transcriptome	New Phytologist	10.1111/nph.19849	PRJNA1078428
Solanum chacoense	Genome	Nature Plants	10.1038/s41477-024-01655-6	http://ibi.zju.edu.cn/N3database/index.php
Solanum chacoense	Transcriptome	Plant Communications	10.1016/j.xplc.2023.100595	SRR2782413
Solanum cheesmaniae	Transcriptome	Plant Communications	10.1016/j.xplc.2023.100595	SRR3031981
Solanum chilense	Genome	Nature Plants	10.1038/s41477-024-01655-6	http://ibi.zju.edu.cn/N3database/index.php
Solanum chmielewskii	Genome	Nature Plants	10.1038/s41477-024-01655-6	http://ibi.zju.edu.cn/N3database/index.php
Solanum chmielewskii	Transcriptome	Plant Communications	10.1016/j.xplc.2023.100595	SRR3031967
Solanum chomatophilum	Genome	Nature Plants	10.1038/s41477-024-01655-6	http://ibi.zju.edu.cn/N3database/index.php
Solanum cinnamomeum	Transcriptome	New Phytologist	10.1111/nph.19849	PRJNA1078428
Solanum cladotrichum	Transcriptome	New Phytologist	10.1111/nph.19849	PRJNA1078428
Solanum clarkiae	Transcriptome	Plant Communications	10.1016/j.xplc.2023.100595	PRJNA827705
Solanum coactiliferum	Transcriptome	Plant Communications	10.1016/j.xplc.2023.100595	PRJNA827705
Solanum commersonii	Genome	Nature Plants	10.1038/s41477-024-01655-6	http://ibi.zju.edu.cn/N3database/index.php
Solanum concinnum	Transcriptome	New Phytologist	10.1111/nph.19849	PRJNA1078428
Solanum conocarpum	Transcriptome	New Phytologist	10.1111/nph.19849	PRJNA1078428
Solanum cordovense	Transcriptome	New Phytologist	10.1111/nph.19849	PRJNA1078428
Solanum corneliomuelleri	Transcriptome	Plant Communications	10.1016/j.xplc.2023.100595	SRR3031963
Solanum corneliomulleri	Genome	Nature Plants	10.1038/s41477-024-01655-6	http://ibi.zju.edu.cn/N3database/index.php
Solanum crispum	Transcriptome	Plant Communications	10.1016/j.xplc.2023.100595	PRJNA827705
Solanum cyclophyllum	Transcriptome	New Phytologist	10.1111/nph.19849	PRJNA1078428
Solanum dasyphyllum	Transcriptome	New Phytologist	10.1111/nph.19849	PRJNA1078428
Solanum decompositiflorum	Transcriptome	New Phytologist	10.1111/nph.19849	PRJNA1078428
Solanum deflexiflorum	Transcriptome	New Phytologist	10.1111/nph.19849	PRJNA1078428
Solanum delagoense	Transcriptome	New Phytologist	10.1111/nph.19849	PRJNA1078428
Solanum didymum	Transcriptome	New Phytologist	10.1111/nph.19849	PRJNA1078428
Solanum diphyllum	Transcriptome	Plant Communications	10.1016/j.xplc.2023.100595	PRJNA827705
Solanum dolosum	Transcriptome	New Phytologist	10.1111/nph.19849	PRJNA1078428

Solanum donianum	Transcriptome	New Phytologist	10.1111/nph.19849	PRJNA1078428
Solanum douglasii	Transcriptome	Plant Communications	10.1016/j.xplc.2023.100595	PRJNA827705
Solanum dulcamara	Genome	Cell	10.1016/j.cell.2023.04.008	PRJNA839598
Solanum dulcamara	Transcriptome	Plant Communications	10.1016/j.xplc.2023.100595	ERR2040627
Solanum elaeagnifolium	Transcriptome	Plant Communications	10.1016/j.xplc.2023.100595	SRR2038442
Solanum enantiophyllanthum	Transcriptome	New Phytologist	10.1111/nph.19849	PRJNA1078428
Solanum ensifolium	Transcriptome	New Phytologist	10.1111/nph.19849	PRJNA1078428
Solanum erianthum	Genome	Cell	10.1016/j.cell.2023.04.008	PRJNA839598
Solanum erianthum	Transcriptome	Plant Communications	10.1016/j.xplc.2023.100595	PRJNA827705
Solanum etuberosum	Genome	Nature Plants	10.1038/s41477-024-01655-6	http://ibi.zju.edu.cn/N3database/index.php
Solanum evolvuloides	Genome	New Phytologist	10.1111/nph.19849	PRJNA1078428
Solanum furcatum	Transcriptome	Plant Communications	10.1016/j.xplc.2023.100595	PRJNA827705
Solanum galapagense	Genome	Nature Plants	10.1038/s41477-024-01655-6	http://ibi.zju.edu.cn/N3database/index.php
Solanum galapagense	Transcriptome	Plant Communications	10.1016/j.xplc.2023.100595	SRR3031961
Solanum gnaphalocarpon	Transcriptome	New Phytologist	10.1111/nph.19849	PRJNA1078428
Solanum granuloso-leprosum	Transcriptome	New Phytologist	10.1111/nph.19849	PRJNA1078428
Solanum guineense	Transcriptome	Plant Communications	10.1016/j.xplc.2023.100595	PRJNA827705
Solanum habrochaites	Genome	Nature Plants	10.1038/s41477-024-01655-6	http://ibi.zju.edu.cn/N3database/index.php
Solanum heteropodium	Transcriptome	Plant Communications	10.1016/j.xplc.2023.100595	PRJNA827705
Solanum hexandrum	Transcriptome	New Phytologist	10.1111/nph.19849	PRJNA1078428
Solanum hutchisonii	Genome	New Phytologist	10.1111/nph.19849	PRJNA1078428
Solanum incanum	Transcriptome	Plant Communications	10.1016/j.xplc.2023.100595	SRR2289250
Solanum incarceratum	Transcriptome	New Phytologist	10.1111/nph.19849	PRJNA1078428
Solanum incurvum	Transcriptome	New Phytologist	10.1111/nph.19849	PRJNA1078428
Solanum inodorum	Transcriptome	New Phytologist	10.1111/nph.19849	PRJNA1078428
Solanum insidiosum	Transcriptome	New Phytologist	10.1111/nph.19849	PRJNA1078428
Solanum isodynamum	Transcriptome	New Phytologist	10.1111/nph.19849	PRJNA1078428
Solanum itatiaiaea	Transcriptome	New Phytologist	10.1111/nph.19849	PRJNA1078428
Solanum jamaicense	Transcriptome	New Phytologist	10.1111/nph.19849	PRJNA1078428
Solanum jamesii	Genome	Nature Plants	10.1038/s41477-024-01655-6	http://ibi.zju.edu.cn/N3database/index.php
Solanum kriegeri	Transcriptome	New Phytologist	10.1111/nph.19849	PRJNA1078428
Solanum lacerdae	Transcriptome	New Phytologist	10.1111/nph.19849	PRJNA1078428
Solanum laciniatum	Genome	Cell	10.1016/j.cell.2023.04.008	PRJNA839598
Solanum laciniatum	Transcriptome	Plant Communications	10.1016/j.xplc.2023.100595	PRJNA827705
Solanum lasiocarpum	Transcriptome	Plant Communications	10.1016/j.xplc.2023.100595	PRJNA827705
Solanum latiflorum	Transcriptome	New Phytologist	10.1111/nph.19849	PRJNA1078428
Solanum laurifrons	Transcriptome	New Phytologist	10.1111/nph.19849	PRJNA1078428
Solanum laxum	Transcriptome	Plant Communications	10.1016/j.xplc.2023.100595	PRJNA827705
Solanum leopoldense	Transcriptome	Plant Communications	10.1016/j.xplc.2023.100595	PRJNA827705
Solanum leucocarpon	Genome	New Phytologist	10.1111/nph.19849	PRJNA1078428
Solanum lidii	Transcriptome	New Phytologist	10.1111/nph.19849	PRJNA1078428
Solanum lignicaule	Genome	Nature Plants	10.1038/s41477-024-01655-6	http://ibi.zju.edu.cn/N3database/index.php
Solanum ligustrinum	Transcriptome	New Phytologist	10.1111/nph.19849	PRJNA1078428
Solanum linnaeanum	Transcriptome	New Phytologist	10.1111/nph.19849	PRJNA1078428
Solanum longifilamentum	Transcriptome	New Phytologist	10.1111/nph.19849	PRJNA1078428
Solanum lycocarpum	Transcriptome	New Phytologist	10.1111/nph.19849	PRJNA1078428
Solanum lycopersicoides	Genome	Nature Plants	10.1038/s41477-024-01655-6	http://ibi.zju.edu.cn/N3database/index.php
Solanum lycopersicum	Genome	Nature Plants	10.1038/s41477-024-01655-6	http://ibi.zju.edu.cn/N3database/index.php
Solanum lyratum	Genome	Cell	10.1016/j.cell.2023.04.008	PRJNA839598
Solanum macrocarpon	Genome	Cell	10.1016/j.cell.2023.04.008	PRJNA839598
Solanum macrotonum	Transcriptome	New Phytologist	10.1111/nph.19849	PRJNA1078428
Solanum mammosum	Genome	Cell	10.1016/j.cell.2023.04.008	PRJNA839598
Solanum mammosum	Transcriptome	Plant Communications	10.1016/j.xplc.2023.100595	PRJNA827705
Solanum mapiriense	Genome	New Phytologist	10.1111/nph.19849	PRJNA1078428
Solanum mauritianum	Transcriptome	New Phytologist	10.1111/nph.19849	PRJNA1078428
Solanum melongena	Genome	Nature Plants	10.1038/s41477-024-01655-6	http://ibi.zju.edu.cn/N3database/index.php
Solanum metrobotryon	Transcriptome	New Phytologist	10.1111/nph.19849	PRJNA1078428
Solanum morelliforme	Genome	Nature Plants	10.1038/s41477-024-01655-6	http://ibi.zju.edu.cn/N3database/index.php
Solanum multifidum	Transcriptome	New Phytologist	10.1111/nph.19849	PRJNA1078428
Solanum multiinterruptum	Genome	Nature Plants	10.1038/s41477-024-01655-6	http://ibi.zju.edu.cn/N3database/index.php
Solanum muricatum	Genome	Nature Plants	10.1038/s41477-024-01655-6	http://ibi.zju.edu.cn/N3database/index.php
Solanum muricatum	Transcriptome	Plant Communications	10.1016/j.xplc.2023.100595	SRR2239214
Solanum neorickii	Genome	Nature Plants	10.1038/s41477-024-01655-6	http://ibi.zju.edu.cn/N3database/index.php
Solanum neorickii	Transcriptome	Plant Communications	10.1016/j.xplc.2023.100595	SRR3031975
Solanum neorossii	Genome	Nature Plants	10.1038/s41477-024-01655-6	http://ibi.zju.edu.cn/N3database/index.php
Solanum nigrescens	Transcriptome	New Phytologist	10.1111/nph.19849	PRJNA1078428
Solanum nigrum	Transcriptome	Plant Communications	10.1016/j.xplc.2023.100595	SRR2229595
Solanum ochranthum	Genome	Cell	10.1016/j.cell.2023.04.008	PRJNA839598
Solanum ochranthum	Transcriptome	New Phytologist	10.1111/nph.19849	PRJNA1078428
Solanum okadae	Genome	Nature Plants	10.1038/s41477-024-01655-6	http://ibi.zju.edu.cn/N3database/index.php
Solanum oxapampense	Transcriptome	New Phytologist	10.1111/nph.19849	PRJNA1078428
Solanum palinacanthum	Transcriptome	New Phytologist	10.1111/nph.19849	PRJNA1078428
Solanum palustre	Genome	Nature Plants	10.1038/s41477-024-01655-6	http://ibi.zju.edu.cn/N3database/index.php
Solanum panduriforme	Transcriptome	New Phytologist	10.1111/nph.19849	PRJNA1078428
Solanum paniculatum	Transcriptome	New Phytologist	10.1111/nph.19849	PRJNA1078428
Solanum paucidens	Transcriptome	New Phytologist	10.1111/nph.19849	PRJNA1078428
Solanum paucissectum	Genome	Nature Plants	10.1038/s41477-024-01655-6	http://ibi.zju.edu.cn/N3database/index.php
Solanum pennellii	Genome	Nature Plants	10.1038/s41477-024-01655-6	http://ibi.zju.edu.cn/N3database/index.php
Solanum peruvianum	Genome	Nature Plants	10.1038/s41477-024-01655-6	http://ibi.zju.edu.cn/N3database/index.php
Solanum peruvianum	Transcriptome	Plant Communications	10.1016/j.xplc.2023.100595	SRR3031977
Solanum phaseoloides	Genome	New Phytologist	10.1111/nph.19849	PRJNA1078428
Solanum phureja	Genome	Nature Plants	10.1038/s41477-024-01655-6	http://ibi.zju.edu.cn/N3database/index.php
Solanum phureja x Solanum tuberosum	Genome	Nature Plants	10.1038/s41477-024-01655-6	http://ibi.zju.edu.cn/N3database/index.php
Solanum piluliferum	Transcriptome	New Phytologist	10.1111/nph.19849	PRJNA1078428
Solanum pimpinellifolium	Genome	Nature Plants	10.1038/s41477-024-01655-6	http://ibi.zju.edu.cn/N3database/index.php
Solanum pinnatisectum	Genome	Nature Plants	10.1038/s41477-024-01655-6	http://ibi.zju.edu.cn/N3database/index.php
Solanum piurae	Genome	Nature Plants	10.1038/s41477-024-01655-6	http://ibi.zju.edu.cn/N3database/index.php
Solanum polygamum	Transcriptome	New Phytologist	10.1111/nph.19849	PRJNA1078428
Solanum polytrichum	Genome	New Phytologist	10.1111/nph.19849	PRJNA1078428
Solanum pseudocapsicum	Genome	Cell	10.1016/j.cell.2023.04.008	PRJNA839598
Solanum pseudocapsicum	Transcriptome	Plant Communications	10.1016/j.xplc.2023.100595	PRJNA827705
Solanum pseudoquina	Transcriptome	New Phytologist	10.1111/nph.19849	PRJNA1078428
Solanum pyracanthos	Transcriptome	New Phytologist	10.1111/nph.19849	PRJNA1078428
Solanum quadriloculatum	Transcriptome	Plant Communications	10.1016/j.xplc.2023.100595	PRJNA827705
Solanum quitoense	Transcriptome	Plant Communications	10.1016/j.xplc.2023.100595	SRR2229599
Solanum reptans	Transcriptome	New Phytologist	10.1111/nph.19849	PRJNA1078428

Solanum retroflexum	Genome	Cell	10.1016/j.cell.2023.04.008	PRJNA839598
Solanum rhaphiotes	Transcriptome	Plant Communications	10.1016/j.xplc.2023.100595	PRJNA827705
Solanum riparium	Transcriptome	New Phytologist	10.1111/nph.19849	PRJNA1078428
Solanum robustum	Transcriptome	New Phytologist	10.1111/nph.19849	PRJNA1078428
Solanum rostratum	Genome	Nature Plants	10.1038/s41477-024-01655-6	http://ibi.zju.edu.cn/N3database/index.php
Solanum rostratum	Transcriptome	Plant Communications	10.1016/j.xplc.2023.100595	PRJNA827705
Solanum rugosum	Transcriptome	New Phytologist	10.1111/nph.19849	PRJNA1078428
Solanum sanctae-catharinae	Transcriptome	New Phytologist	10.1111/nph.19849	PRJNA1078428
Solanum saponaceum	Transcriptome	New Phytologist	10.1111/nph.19849	PRJNA1078428
Solanum scabrum	Genome	Cell	10.1016/j.cell.2023.04.008	PRJNA839598
Solanum scabrum	Transcriptome	Plant Communications	10.1016/j.xplc.2023.100595	PRJNA827705
Solanum sciadostylis	Transcriptome	New Phytologist	10.1111/nph.19849	PRJNA1078428
Solanum scuticum	Transcriptome	New Phytologist	10.1111/nph.19849	PRJNA1078428
Solanum seaforthianum	Genome	Cell	10.1016/j.cell.2023.04.008	PRJNA839598
Solanum seaforthianum	Transcriptome	Plant Communications	10.1016/j.xplc.2023.100595	PRJNA827705
Solanum sejunctum	Transcriptome	Plant Communications	10.1016/j.xplc.2023.100595	PRJNA827705
Solanum sellowianum	Transcriptome	New Phytologist	10.1111/nph.19849	PRJNA1078428
Solanum semotum	Transcriptome	New Phytologist	10.1111/nph.19849	PRJNA1078428
Solanum septemlobum	Genome	Cell	10.1016/j.cell.2023.04.008	PRJNA839598
Solanum septemlobum	Transcriptome	Plant Communications	10.1016/j.xplc.2023.100595	PRJNA827705
Solanum sessile	Transcriptome	New Phytologist	10.1111/nph.19849	PRJNA1078428
Solanum sessiliflorum	Transcriptome	New Phytologist	10.1111/nph.19849	PRJNA1078428
Solanum sinuatirecurvum	Transcriptome	Plant Communications	10.1016/j.xplc.2023.100595	PRJNA827705
Solanum sisymbriifolium	Genome	Cell	10.1016/j.cell.2023.04.008	PRJNA839598
Solanum sisymbriifolium	Transcriptome	Plant Communications	10.1016/j.xplc.2023.100595	ERR2040632
Solanum sitiens	Genome	Nature Plants	10.1038/s41477-024-01655-6	http://ibi.zju.edu.cn/N3database/index.php
Solanum sitiens	Transcriptome	Plant Communications	10.1016/j.xplc.2023.100595	SRR3031985
Solanum sogarandinum	Genome	Nature Plants	10.1038/s41477-024-01655-6	http://ibi.zju.edu.cn/N3database/index.php
Solanum sotobosquense	Transcriptome	New Phytologist	10.1111/nph.19849	PRJNA1078428
Solanum sp nov.	Transcriptome	New Phytologist	10.1111/nph.19849	PRJNA1078428
Solanum spirale	Genome	Cell	10.1016/j.cell.2023.04.008	PRJNA839598
Solanum stenotomum	Genome	Nature Plants	10.1038/s41477-024-01655-6	http://ibi.zju.edu.cn/N3database/index.php
Solanum stramoniifolium	Genome	Cell	10.1016/j.cell.2023.04.008	PRJNA839598
Solanum subinerme	Transcriptome	New Phytologist	10.1111/nph.19849	PRJNA1078428
Solanum subtusviolaceum	Transcriptome	New Phytologist	10.1111/nph.19849	PRJNA1078428
Solanum subumbellatum	Transcriptome	New Phytologist	10.1111/nph.19849	PRJNA1078428
Solanum succosum	Transcriptome	Plant Communications	10.1016/j.xplc.2023.100595	PRJNA827705
Solanum swartzianum	Transcriptome	New Phytologist	10.1111/nph.19849	PRJNA1078428
Solanum sycophanta	Transcriptome	New Phytologist	10.1111/nph.19849	PRJNA1078428
Solanum thelopodium	Transcriptome	New Phytologist	10.1111/nph.19849	PRJNA1078428
Solanum tomentosum	Transcriptome	New Phytologist	10.1111/nph.19849	PRJNA1078428
Solanum torvum	Genome	Cell	10.1016/j.cell.2023.04.008	PRJNA839598
Solanum torvum	Transcriptome	Plant Communications	10.1016/j.xplc.2023.100595	PRJNA827705
Solanum trachycyphum	Transcriptome	New Phytologist	10.1111/nph.19849	PRJNA1078428
Solanum trizygum	Transcriptome	New Phytologist	10.1111/nph.19849	PRJNA1078428
Solanum tuberosum	Genome	Nature Plants	10.1038/s41477-024-01655-6	http://ibi.zju.edu.cn/N3database/index.php
Solanum uleanum	Transcriptome	New Phytologist	10.1111/nph.19849	PRJNA1078428
Solanum umbelliferum var. glabrescens	Transcriptome	Plant Communications	10.1016/j.xplc.2023.100595	PRJNA827705
Solanum umbelliferum var. xanti	Transcriptome	Plant Communications	10.1016/j.xplc.2023.100595	PRJNA827705
Solanum undatum	Transcriptome	Plant Communications	10.1016/j.xplc.2023.100595	PRJNA827705
Solanum uporo	Transcriptome	New Phytologist	10.1111/nph.19849	PRJNA1078428
Solanum vaillantii	Transcriptome	New Phytologist	10.1111/nph.19849	PRJNA1078428
Solanum variabile	Transcriptome	New Phytologist	10.1111/nph.19849	PRJNA1078428
Solanum velleum	Transcriptome	New Phytologist	10.1111/nph.19849	PRJNA1078428
Solanum velutinum	Transcriptome	New Phytologist	10.1111/nph.19849	PRJNA1078428
Solanum venosum	Transcriptome	New Phytologist	10.1111/nph.19849	PRJNA1078428
Solanum verecundum	Transcriptome	New Phytologist	10.1111/nph.19849	PRJNA1078428
Solanum vernei	Genome	Nature Plants	10.1038/s41477-024-01655-6	http://ibi.zju.edu.cn/N3database/index.php
Solanum verrucosum	Genome	Nature Plants	10.1038/s41477-024-01655-6	http://ibi.zju.edu.cn/N3database/index.php
Solanum vespertilio	Transcriptome	New Phytologist	10.1111/nph.19849	PRJNA1078428
Solanum viarum	Genome	Cell	10.1016/j.cell.2023.04.008	PRJNA839598
Solanum violaceum	Transcriptome	New Phytologist	10.1111/nph.19849	PRJNA1078428
Solanum volubile	Transcriptome	New Phytologist	10.1111/nph.19849	PRJNA1078428
Solanum wendlandii	Transcriptome	New Phytologist	10.1111/nph.19849	PRJNA1078428
Solanum woodburyi	Transcriptome	New Phytologist	10.1111/nph.19849	PRJNA1078428
Solanum wrightii	Genome	Cell	10.1016/j.cell.2023.04.008	PRJNA839598
Solanum wrightii	Transcriptome	Plant Communications	10.1016/j.xplc.2023.100595	PRJNA827705
Streptosolen jamesonii	Transcriptome	Plant Communications	10.1016/j.xplc.2023.100595	PRJNA827705
Trianaea nobilis	Genome	Plant Communications	10.1016/j.xplc.2023.100595	PRJNA827705
Tubocapsicum anomalum	Genome	Cell	10.1016/j.cell.2023.04.008	PRJNA839598
Tubocapsicum anomalum	Transcriptome	Plant Communications	10.1016/j.xplc.2023.100595	PRJNA827705
Vassobia breviflora	Transcriptome	Plant Communications	10.1016/j.xplc.2023.100595	PRJNA827705
Vestia foetida	Transcriptome	Plant Communications	10.1016/j.xplc.2023.100595	PRJNA827705
Withania somnifera	Transcriptome	Plant Communications	10.1016/j.xplc.2023.100595	PRJNA827705
Witheringia meiantha	Transcriptome	Plant Communications	10.1016/j.xplc.2023.100595	PRJNA827705
Witheringia riparia	Transcriptome	Plant Communications	10.1016/j.xplc.2023.100595	PRJNA827705

[1] Information retrived from reference database and phylogenetic studies

Table S5. Viral genera whose representative species were first discovered in Solanaceae hosts

Virus family	Virus genus	Virus species (reference)	Virus species[1] (total)	Virus species (Solanaceae)	Virus_species (Solanaceae, %)
Potyviridae	*Potyvirus*	potato virus Y	200	25	12.50%
Secoviridae	*Nepovirus*	tomato ringspot virus	48	7	14.58%
Secoviridae	*Torradovirus*	tomato torrado virus	8	2	25.00%
Alphaflexiviridae	*Potexvirus*	potato virus X	48	3	6.25%
Virgaviridae	*Tobamovirus*	tobacco mosaic virus	37	13	35.14%
Virgaviridae	*Pomovirus*	potato mop-top virus	5	2	40.00%
Virgaviridae	*Tobravirus*	tobacco rattle virus	3	2	66.67%
Solemoviridae	*Polerovirus*	potato leafroll virus	26	8	30.77%
Tospoviridae	*Orthotospovirus*	tomato spotted wilt virus	26	6	23.08%
Tombusviridae	*Tombusvirus*	tomato bushy stunt virus	13	4	30.77%
Tombusviridae	*Betanecrovirus*	tobacco necrosis virus D	3	1	33.33%
Tombusviridae	*Alphanecrovirus*	tobacco necrosis virus A	4	2	50.00%
Tolecusatellitidae	*Deltasatellite*	tomato leaf curl deltasatellite	12	3	25.00%
Pospiviroidae	*Pospiviroid*	potato spindle tuber viroid	10	5	50.00%
Amalgaviridae	*Amalgavirus*	southern tomato virus	9	1	11.11%
Betaflexiviridae	*Tepovirus*	potato virus T	5	1	20.00%
Partitiviridae	*Deltapartitivirus*	pepper cryptic virus 1	5	2	40.00%
Unassigned	*Albetovirus*	satellite tobacco necrosis virus 1	3	3	100.00%
Unassigned	*Virtovirus*	satellite tobacco mosaic virus	1	1	100.00%
Caulimoviridae	*Solendovirus*	tobacco vein clearing virus	2	1	50.00%
Caulimoviridae	*Petuvirus*	petunia vein clearing virus	1	1	100.00%
Geminiviridae	*Topilevirus*	tomato apical leaf curl virus	2	2	100.00%
Geminiviridae	*Topocuvirus*	tomato pseudo-curly top virus	1	1	100.00%
Avsunviroidae	*Elaviroid*	eggplant latent viroid	1	1	100.00%

[1] Numbers from the International Committee for the Taxonomy of Viruses, virus metadata resource VMR_MSL38_v2

Table S6. Cumulative numbers of viral species per plant family of first discovery

Plant family	Virus genera[1] (number)	Virus species[1] (number)	*Z*	Grubbs' test
**Solanaceae**	52	366	7.36	*P* < 0.01
Fabaceae	59	226	4.4	n.s.
Poaceae	46	170	3.21	n.s.
Malvaceae	18	160	3	n.s.
Asteraceae	39	116	2.07	n.s.
Rosaceae	48	113	2	n.s.
Cucurbitaceae	29	79	1.28	n.s.
Euphorbiaceae	17	53	0.73	n.s.
Vitaceae	20	49	0.65	n.s.
Amaryllidaceae	12	45	0.56	n.s.
Apiaceae	21	40	0.46	n.s.
Brassicaceae	23	39	0.44	n.s.
Convolvulaceae	12	38	0.42	n.s.
Amaranthaceae	21	38	0.42	n.s.
Rutaceae	20	31	0.27	n.s.
Orchidaceae	14	30	0.25	n.s.
Lamiaceae	16	28	0.2	n.s.
Dioscoreaceae	9	23	0.1	n.s.
Oleaceae	13	20	0.04	n.s.
Caricaceae	7	20	0.04	n.s.
Musaceae	8	19	0.01	n.s.
Asparagaceae	8	17	0.03	n.s.
Passifloraceae	8	16	0.05	n.s.
Apocynaceae	6	16	0.05	n.s.
Cactaceae	7	15	0.07	n.s.
Ericaceae	12	14	0.09	n.s.
Liliaceae	6	13	0.11	n.s.
Arecaceae	8	13	0.11	n.s.
Araceae	7	13	0.11	n.s.
Ranunculaceae	6	12	0.13	n.s.
Moraceae	9	12	0.13	n.s.
Caryophyllaceae	11	11	0.16	n.s.
Cannabaceae	8	11	0.16	n.s.
Acanthaceae	2	10	0.18	n.s.
Iridaceae	4	9	0.2	n.s.
Theaceae	7	8	0.22	n.s.
Plantaginaceae	8	8	0.22	n.s.
Grossulariaceae	8	8	0.22	n.s.
Actinidiaceae	6	8	0.22	n.s.
Zingiberaceae	4	7	0.24	n.s.
Nyctaginaceae	6	7	0.24	n.s.
Geraniaceae	4	7	0.24	n.s.
Caprifoliaceae	4	7	0.24	n.s.
Bromeliaceae	3	7	0.24	n.s.
Onagraceae	2	6	0.26	n.s.
Adoxaceae	4	6	0.26	n.s.
Verbenaceae	3	5	0.28	n.s.
Urticaceae	2	5	0.28	n.s.
Rubiaceae	5	5	0.28	n.s.
Ebenaceae	4	5	0.28	n.s.
Campanulaceae	5	5	0.28	n.s.
Zosteraceae	3	4	0.3	n.s.
Sapindaceae	4	4	0.3	n.s.

Crassulaceae	4	4	0.3 n.s.
Basellaceae	4	4	0.3 n.s.
Alstroemeriaceae	3	4	0.3 n.s.
Thymelaeaceae	2	3	0.32 n.s.
Scrophulariaceae	2	3	0.32 n.s.
Polemoniaceae	1	3	0.32 n.s.
Orobanchaceae	3	3	0.32 n.s.
Melanthiaceae	2	3	0.32 n.s.
Lauraceae	3	3	0.32 n.s.
Gentianaceae	3	3	0.32 n.s.
Commelinaceae	2	3	0.32 n.s.
Cleomaceae	2	3	0.32 n.s.
Cannaceae	2	3	0.32 n.s.
Araliaceae	2	3	0.32 n.s.
Aquifoliaceae	3	3	0.32 n.s.
Salicaceae	2	2	0.35 n.s.
Rhamnaceae	2	2	0.35 n.s.
Polygonaceae	2	2	0.35 n.s.
Papaveraceae	2	2	0.35 n.s.
Oxalidaceae	2	2	0.35 n.s.
Linderniaceae	2	2	0.35 n.s.
Hydrangeaceae	2	2	0.35 n.s.
Gesneriaceae	2	2	0.35 n.s.
Fagaceae	2	2	0.35 n.s.
Cycadaceae	2	2	0.35 n.s.
Colchicaceae	1	2	0.35 n.s.
Chenopodiaceae	2	2	0.35 n.s.
Celastraceae	1	2	0.35 n.s.
Betulaceae	2	2	0.35 n.s.
Balsaminaceae	2	2	0.35 n.s.
Anacardiaceae	2	2	0.35 n.s.
Viscaceae	1	1	0.37 n.s.
Violaceae	1	1	0.37 n.s.
Ulmaceae	1	1	0.37 n.s.
Tropaeolaceae	1	1	0.37 n.s.
Schisandraceae	1	1	0.37 n.s.
Primulaceae	1	1	0.37 n.s.
Portulacaceae	1	1	0.37 n.s.
Polygalaceae	1	1	0.37 n.s.
Plumbaginaceae	1	1	0.37 n.s.
Piperaceae	1	1	0.37 n.s.
Pinaceae	1	1	0.37 n.s.
Phytolaccaceae	1	1	0.37 n.s.
Phyllanthaceae	1	1	0.37 n.s.
Pedaliaceae	1	1	0.37 n.s.
Nymphaeaceae	1	1	0.37 n.s.
Montiaceae	1	1	0.37 n.s.
Meliaceae	1	1	0.37 n.s.
Lythraceae	1	1	0.37 n.s.
Juncaceae	1	1	0.37 n.s.
Juglandaceae	1	1	0.37 n.s.
Hypericaceae	1	1	0.37 n.s.
Hydrocharitaceae	1	1	0.37 n.s.
Hyacinthaceae	1	1	0.37 n.s.
Garryaceae	1	1	0.37 n.s.
Dryopteridaceae	1	1	0.37 n.s.

Dilleniaceae	1	1	0.37 n.s.
Costaceae	1	1	0.37 n.s.
Corynocarpaceae	1	1	0.37 n.s.
Capparaceae	1	1	0.37 n.s.
Begoniaceae	1	1	0.37 n.s.
Azollaceae	1	1	0.37 n.s.

[1] Numbers from the International Committee for the Taxonomy of Viruses, virus metadata resource VMR_MSL38_v2
